# Structure and Properties of Composite Fibers Based on Chitosan and Single-Walled Carbon Nanotubes for Peripheral Nerve Regeneration

**DOI:** 10.3390/polym15132860

**Published:** 2023-06-28

**Authors:** Elena N. Dresvyanina, Nurjemal A. Tagandurdyyeva, Vera V. Kodolova-Chukhontseva, Irina P. Dobrovol’skaya, Almaz M. Kamalov, Yulia A. Nashchekina, Alexey V. Nashchekin, Alexey G. Ivanov, Galina Yu. Yukina, Vladimir E. Yudin

**Affiliations:** 1 Institute of Textile and Fashion, Saint Petersburg State University of Industrial Technologies and Design, B. Morskaya Str., 18, Saint Petersburg 191186, Russia; 2 Institute of Biomedical Systems and Biotechnology, Peter the Great Saint Petersburg Polytechnic University, Polytekhnicheskaya Str., 29, Saint Petersburg 195251, Russia; jemala_96@mail.ru (N.A.T.); vera_kodolova@mail.ru (V.V.K.-C.); spb.kamalov@gmail.com (A.M.K.); 3 Institute of Macromolecular Compounds of Russian Academy of Sciences, VO Bolshoy pr., 31, Saint Petersburg 199004, Russia; zair2@mail.ru (I.P.D.); alexey.ivanov@bk.ru (A.G.I.); yudinve@gmail.com (V.E.Y.); 4 Institute of Cytology Russian Academy of Sciences, Tikhoretsky Ave., 4, Saint Petersburg 194064, Russia; nashchekina.yu@mail.ru; 5 Ioffe Institute, Polytekhnicheskaya Str., 26, Saint Petersburg 194021, Russia; nashchekin@mail.ioffe.ru; 6 Pavlov First Saint Petersburg State Medical University, L’va Tolstogo Str. 6-8, Saint Petersburg 197022, Russia; pipson@inbox.ru

**Keywords:** chitosan, wet spinning, single-wall carbon nanotubes, composites, peripheral nerve regeneration

## Abstract

This study focused on a potential application of electrically conductive, biocompatible, bioresorbable fibers for tubular conduits aimed at the regeneration of peripheral nerves. The conducting, mechanical, and biological properties of composite fibers based on chitosan and single-walled carbon nanotubes were investigated in this paper. It was shown that introducing 0.5 wt.% of SWCNT into the composite fibers facilitated the formation of a denser fiber structure, resulting in improved strength (σ = 260 MPa) and elastic (E = 14 GPa) characteristics. Additionally, the composite fibers were found to be biocompatible and did not cause significant inflammation or deformation during in vivo studies. A thin layer of connective tissue formed around the fiber.

## 1. Introduction

One of the main tasks of tissue engineering is the development of scaffolds based on biocompatible and bioresorbable polymers that can be implanted into a living organism; after implantation, these scaffolds (surrounded by biological media) stimulate cell proliferative activity, which, in turn, promotes the regeneration of damaged tissue. As a result, a new tissue identical to that of the recipient is formed [1,2,3,4,5]. The ability of stem cells to proliferate, grow, and differentiate is determined by electrical signals that take part in cell communications and behavior and, correspondingly, in tissue regeneration processes [6,7,8]. This role of electrical signals is especially significant in the regeneration of peripheral nerves [6,8,9,10], bone, cartilage, and muscular tissues [1,2,3,11]. Therefore, the scaffolds should possess a certain level of electrical conductivity (close to the electrical characteristics of living tissues and cells [4,12]) to enhance their biocompatibility, modulate cell/tissue responses, and facilitate cell stimulation [6,7,13]. The main factor that should be considered in the development of conducting scaffolds (which are intended to contact living cells and tissues) is the mechanism of the combined action of the electrical signals caused by the electrical conductivity of the materials and the signals of ionic conductivity received by the cells.

The scaffolds should also possess necessary mechanical strength and deformation characteristics that are similar to those of native tissues.

An important field of application of bioresorbable conducting materials is their use as the main elements in conduits, which are the structures intended for the stimulation of regenerative processes in peripheral nerves [7,12,14,15,16,17,18,19].

Relatively short fragments of damaged peripheral nerves (up to 3 cm) are reconstructed via surgical methods without the use of implants or transplants. However, when direct suturing is impossible (because the resulting tension affects nerve regeneration), transplants or implants in the form of tubular guide conduits are used to support regeneration [20,21].

The reconstruction of peripheral nerves is most often performed via autotransplantation, which is considered the “gold standard” [22,23,24,25,26] since the patient’s organism serves as the source of the transplanted material. An autotransplant is a non-immunogenic scaffold that provides a regenerating nerve with viable Schwann cells and the corresponding neurotrophic factors [25,27,28]. However, this method has numerous limitations relating to the necessity of multiple surgeries, differences in sizes and structures between transplants and nerve tissue, the invasion of scar tissue, an absence of donor material, inadequate functional recovery, and aberrant regeneration [24]. These problems can be partially solved through the use of allotransplants and xenotransplants; however, these materials may cause immune rejection, secondary infection, and other systemic side effects that restrict their clinical applications [29].

As a rule, nerve conduits based on natural and synthetic bioresorbable polymers do not suffer from the disadvantages listed above. These conduits are tubular constructions containing guiding elements for neurite growth. Tubular implants facilitate accelerated nerve regeneration by directing neurite growth from the proximal nerve end to its distal end [30,31]. An active search is currently being carried out for modifiers (fillers) that will guide regenerated neurites and accelerate the regeneration process. All types of guides can be divided into biochemical and physical objects. Biochemical guides include Schwann cells, nerve stem cells, mesenchymal stem cells, embryonal stem cells, growth factors, neurotrophic factors, cells, nucleic acids, and the molecules of the extracellular matrix, such as collagen, laminin, fibronectin, etc. [32]. Among the physical guides are microfilaments, microfibers, threads, and multichannel structures introduced directly into the channel lumen [26,33,34,35,36,37,38,39]. These guides simulate the oriented architecture of the fascicular nerve by dividing the lumen into smaller guiding tubes. As a result, neurite dispersion (which usually arises in hollow channels) may be diminished. In addition, the guides exert an influence on the transport of nutrients and the infiltration of blood vessels and restrict the infiltration of cells that may prevent neurite expansion [40,41].

The main advantage of introducing fibrous guides into the lumen is that the combination of a porous structure, large surface area, and topographic signals is an ideal environment for cell attachment and growth [32].

A preparation technique for tubular scaffolds with various geometrical parameters (length, internal diameter, and thickness) based on the production of nano- and microfibers of bioresorbable polymer poly(L-lactide) via electrospinning was previously proposed [42]. Researchers have suggested using mono- and multifilament threads of chitosan obtained via wet spinning as guides [43,44,45] since chitosan possesses several positive medical properties such as biocompatibility, bioresorption capability, the absence of cytotoxicity, and a low environmental impact during processing [46].

However, chitosan fibers do not generally possess electrical conductivity. As mentioned above, to accelerate tissue regeneration, scaffolds should be able to transmit electrical signals of various intensities and frequencies to cellular structures. One way to improve the functional characteristics of chitosan filaments and facilitate better cell communication and tissue responses consists of the preparation of composite materials containing an electrically conductive polymer or filler, specifically, carbon nanoparticles.

It has been demonstrated [47] that the introduction of single-walled carbon nanotubes (SWCNTs) into chitosan scaffolds resulted in the ordering of the polymer structures and increases in the elastic moduli and conductivities of the composite films. The chitosan-based films containing up to 2.5 wt.% of single-walled nanotubes were compatible with a culture of human dermal fibroblasts. The data on the ionic and electronic components of the conductivities of the composite films were reported [47].

Although numerous publications devoted to the preparation and study of the structures and properties of biocompatible conducting polymers and composite materials have appeared in recent years, the knowledge of the preparation techniques and electrophysical properties of these materials (and especially fibers) is not sufficient. On the other hand, it is known that the strength and elastic properties of polymers are fully realized in the oriented state, particularly in fibers.

The aims of the present work included the preparation of electrically conductive composite fibers based on bioresorbable polymer chitosan and single-walled carbon nanotubes, studies of their structure, electrical, strength, and deformation characteristics, and an investigation of their biocompatibility. It is expected that the obtained results will help solve an important practical problem: the preparation of electrically conductive, biocompatible, bioresorbable fibers for tubular conduits, which will be used in the regeneration of peripheral nerves.

## 2. Materials and Methods

Nanofiber-based tubes can be obtained via electrospinning and microbraiding [48,49,50,51]. The porous structures of tubular implants intended for the regeneration of peripheral nerves should prevent the diffusion of connective and muscular tissue into the diastasis area and promote the vascularization of implant walls. Their lumens should contain anisodiametric pores with diameters comparable to those of the neurites of the damaged nerve.

Poly(L-lactide) (PLA) (Purasorb PL10 (Corbion Purac, Amsterdam, The Netherlands)) was used in the preparation of tubular scaffolds. Using an injection pump, a PLA solution in trichloromethane (15%) was fed through a die into an electric field with a strength of E = 1.5 × 10^4^ − 4.0 × 10^5^ V/m. Microfibers precipitated on a cylindrical electrode 1.5 mm in diameter; its rotation speed was equal to 1500 rpm, and the distance between electrodes was 0.15 m. The scaffolds fixed on the cylindrical electrode were then heated at 90 °C for 1 h to achieve partial crystallization of the polymer and improve its mechanical properties. The scaffold wall thickness after thermal treatment was equal to 350 µm.

Composite fibers (guides in the tubular scaffold) were prepared via wet spinning [43,44,45] from a mixture of an aqueous dispersion of single-walled carbon nanotubes (SWCNT) and a 4% solution of chitosan in 2% acetic acid. Chitosan with a molecular mass Mm = (1.64 − 2.1) × 10^5^ and deacetylation degree DD = 92% (Biolog Heppe GmbH, Landsberg, Germany) was used. The SWCNTs (diameter: 1.4 ± 0.3 nm; length: 1–5 µm) were purchased from Carbon Chg, Chernogolovka, Russia.

The aqueous suspension containing 0.05–2.5 wt.% of SWCNTs was subjected to ultrasound treatment for 15 min with an IL10–0.63 dispergator (25 kHz, 630 W) [47,52]. Chitosan powder was added to the aqueous suspension in the amount that provided a final concentration in the solution equal to 4 wt.%. The mixture was stirred for 1 h, and concentrated acetic acid was then added to the solution until its concentration in the chitosan/SWCNT mixture was equal to 2%. The composite solution was stirred for 6 h at room temperature. Solutions containing 0.05, 0.5, and 2.5 wt.% of SWCNT to chitosan mass were prepared. The solutions were filtered and deaerated in a vacuum chamber for 24 h at a pressure of 10 kPa. This method of processing makes it possible to obtain a composite with a uniform distribution of particles in the bulk of the polymer [47].

**Figure 1:**
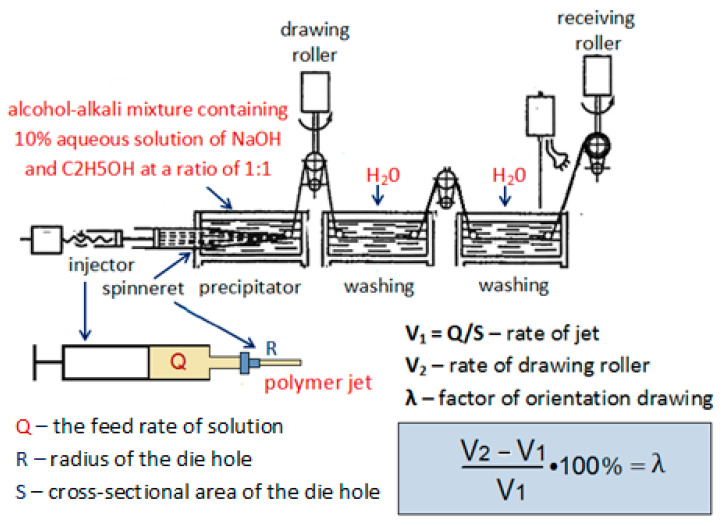
The scheme of preparation of chitosan fibers.

An ethanol/alkali mixture containing C_2_H_5_OH and a 10% aqueous solution of NaOH at a ratio of 1:1 was used as a precipitant in the preparation of the fibers. The solution was extruded using an injection pump through a die 0.6 mm in diameter into the precipitation bath; the feeding rate was 0.2 mL/min, the precipitation time was 150 s, and the values of orientational drawing (λ) varied from 0 to 100%. The prepared fibers were washed with distilled water and dried in air at 50 °C. The scheme of the setup for fiber preparation is presented in Figure 1.

The introduction of guiding fibers into the tubular PLA scaffold was performed immediately before surgery. The guiding fibers were oriented along the direction of neurite growth; the filling of the tubular channel lumen did not exceed 25 vol.%.

The structures of the composite fibers were studied using a JSM-7001F scanning electron microscope (Jeol, Tokyo, Japan). Before measurements were taken, the samples were coated with a gold layer 20 nm thick.

The mechanical properties of the composite fibers were studied with the aid of an Instron 5943 universal testing machine; the loading rate was 10 mm/min, and the base length was 100 mm. Before testing, the fibers were kept in a desiccator for 24 h at a relative air humidity of 66%.

**Figure 2:**
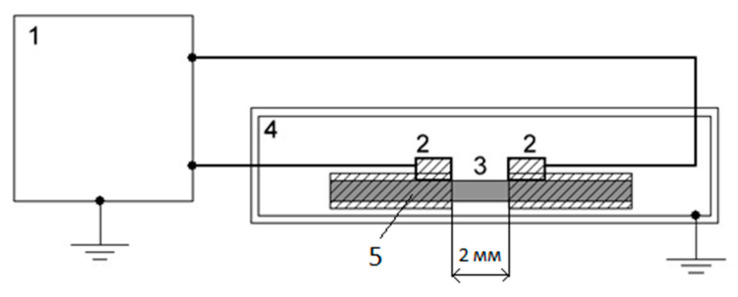
Block diagram of the setup for measuring specific resistivities of fibers. 1—Keithley-6487 Picoammeter/voltage source; 2—copper electrodes; 3—the studied fiber; 4—shielded cell; 5—silver paste.

The specific volume resistivities of the SWCNT-containing chitosan fibers were measured in a constant electric field at voltage U = 100 V with a Keithley-6487 Picoammeter/voltage source under normal pressure, at room temperature, and with an air humidity of 40%. To improve the contact between the samples and electrodes, a silver paint conductive silver paste (SPI, West Chester, PA, USA) was used. The distance between electrodes was 2 mm. The scheme of the setup is presented in Figure 2.

The resistivity measurements of three types of fibers were taken: the initial fibers, the samples kept in a desiccator for 24 h at a relative air humidity of 98%, and the samples exposed to the lysozyme-containing phosphate-buffered saline.

Chitosan fibers are bioresorbable; therefore, the in vitro studies of the resorption of the SWCNT-containing composite fibers were performed to reveal changes in their structures and electrical conductivities.

IR spectra were recorded on a Fourier spectrometer “IRAffinity-1S” (Shimadzu, Kyoto, Japan) in the mid-IR region (700–4000 cm^−1^) using a MIRacle microadapter (PIKE Technologies, Madison, WI, USA) for single attenuated total internal reflection (ATR).

The sterilization of the chitosan samples was carried out under the influence of ozone for 90 min.

The in vitro degradation of the fibers was studied in phosphate-buffered saline (PBS, pH 7.4) containing 1.5 mg/mL of lysozyme (human lysozyme, Sigma-Aldrich, St. Louis, MO, USA) at 37 °C. The lysozyme concentration was chosen according to [53]. The lysozyme solution was refreshed every third day to ensure continuous enzyme activity [54]. After 7, 14, 21, and 28 days, the samples were removed from the media, rinsed with distilled water, dried at 37 °C until a constant weight was reached [55], and weighed. The extent of in vitro degradation was expressed as the percentage of mass lost from the dried fiber after the PBS and lysozyme treatments.

The conductivities of the samples were measured after 14, 21, and 28 days of exposure to the lysozyme-containing phosphate-buffered saline.

The cytotoxicity studies involved human mesenchymal stromal cell lines (FetMSCs). The cell lines were obtained from the Vertebrate Cell Culture Collection (Institute of Cytology RAS, St Petersburg, Russia). The cells were cultivated in a CO_2_ incubator at 37 °C in a humidified atmosphere containing air and 5% CO_2_ in a DMEM nutrient medium (Dulbecco’s modified Eagle’s medium; Gibco) containing 10% (*v*/*v*) of thermally inactivated fetal bovine serum (FBS; HyClone, Logan, UT, USA), 1% of L-glutamine, penicillin (50 units/mL), and 50 µg/mL of streptomycin.

The fiber samples were 2 cm long and were immersed in 2 mL of complete nutrient medium and incubated for 4 days. For the evaluation of cytotoxicity, cells (5.0 × 10^3^ cells/100 µL/well) were seeded into 96-well plates and cultivated for 24 h for attachment. Then, 100 µL of the medium taken after the incubation of the samples was added. The cells cultivated in the standard nutrient medium were used as control samples.

The cells were incubated for 72 h. After the completion of the incubation period, the medium was removed, and the DMEM/F12 medium (50 µL/well) with MTT (0.1 mg/mL) was introduced. The cells were incubated in a CO_2_ incubator for 2 h at 37 °C. After the removal of the supernatant, the formazan crystals formed by metabolically active viable cells were dissolved in dimethylsulfoxide (50 µL/well), and the optical density of the resulting solution was measured at a wavelength of 570 nm with a plate spectrophotometer. The calculations involved a polynomial regression analysis in Microsoft Excel.

In vivo experiments were carried out on male Wistar albino rats, following the rules for working with experimental animals (the principles of the European Convention for the Protection of Vertebrate Animals used for Experimental and other Scientific Purposes, Strasbourg, 1986, and the Declaration of Helsinki of the World Medical Association on Humane animal welfare 1996). The experimental animals weighed between 180 and 200 g, they were 3 months of age, and there were 10 rats in each group. The animals were operated on under general anesthesia (solution containing Zoletil 100, 0.1 mL, and Rometarum, 20 mg/mL: 0.0125 mL per 0.1 kg of animal weight, administered intraperitoneally).

The rat sciatic nerve (the largest nerve in the animal) was selected for nerve defect replacement with polylactide conduits filled with SWCNT-containing chitosan monofibers.

The introduction of guiding fibers into the tubular scaffold was performed immediately before surgery. The diastasis (the distance between the ends of a damaged nerve) was equal to 0.5 cm.

The animals were sacrificed 16 weeks after surgery with CO_2_. For histological studies, fragments of the sciatic nerve with polylactide conduits and chitosan monofibers with different contents of SWCNT were fixed in 10% neutral formalin in phosphate-buffered saline (pH 7.4) for 24 h, dehydrated using a series of ethanol/water solutions with increasing ethanol concentrations, and enclosed in paraffin blocks according to the standard histological technique. To obtain comparable results, the samples were treated simultaneously and under similar conditions. Then, longitudinal cuts of proximal fragments of the conduits and transverse cuts of distal fragments of the nerves (conduit) were obtained. The cuts were 5 µm thick and were prepared with the use of an Accu-Cut SRT 200 microtome (Sakura, Tokyo, Japan), followed by staining with hematoxylin and eosin, using the Picro-Mallory staining technique (BioVitrum, St. Petersburg, Russia). A microscopic analysis of the samples was performed using a Nikon Eclipse E200 light microscope (Nikon, Tokyo, Japan) with 10× ocular and lenses with magnifications of 4, 10, 20, and 40×. Digital images were recorded using a Nikon DS-Fi3 camera (Nikon, Japan).

## 3. Results

### 3.1. Scanning Electron Microscopy

**Figure 3:**
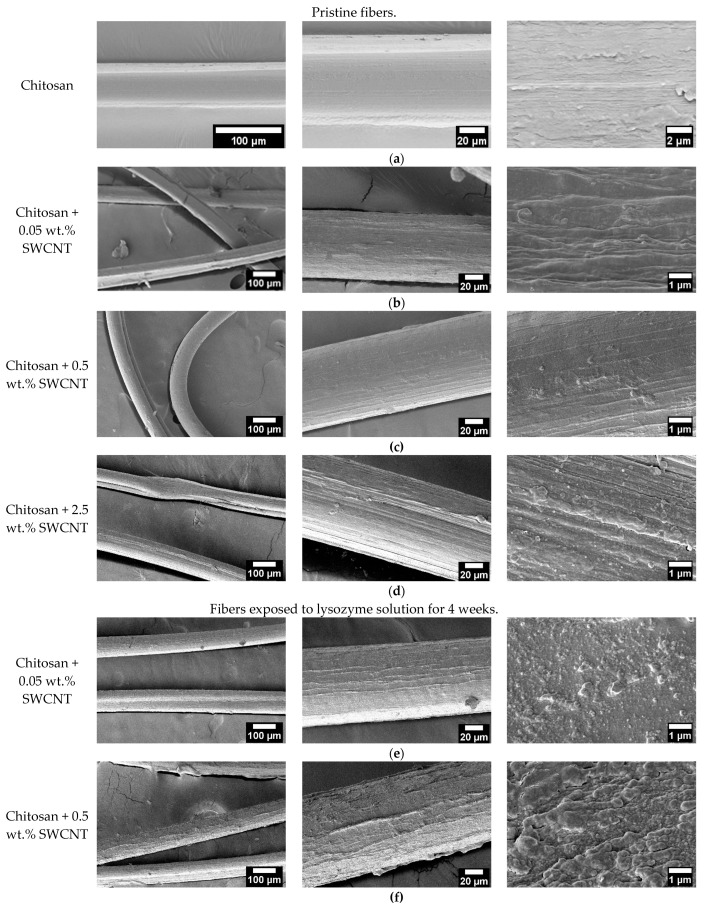

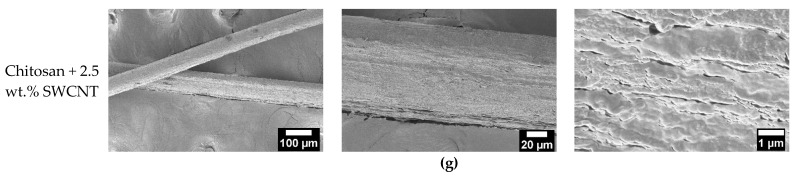
Microphotographs of pure chitosan fibers [43] (**a**), freshly prepared composite fibers containing SWCNTs (0.05% (**b**), 0.5% (**c**), and 2.5 wt.% (**d**)), and the composite fibers containing SWCNTs (0.05% (**e**), 0.5% (**f**), and 2.5 wt. % (**g**)) after exposure to lysozyme solution for 4 weeks.

**Figure 4:**
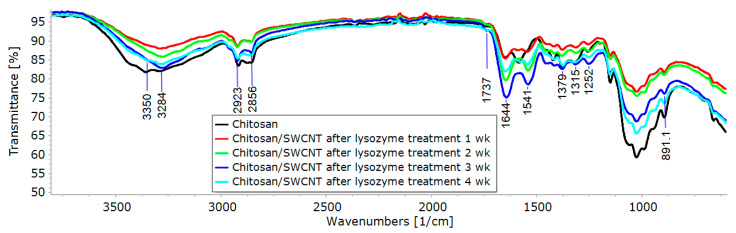
FTIR spectra of chitosan and composite fibers containing 0.5 wt.% SWCNTs treated with lysozyme for 1–4 weeks.

It has been noted [43] that chitosan fibers have smooth surfaces and homogeneous internal structures. The microphotographs of composite fibers presented in Figure 3 indicate that their structures contain lamellar elements, which agrees with the results reported in [45,47]. The addition of extremely small amounts of SWCNTs (0.05 wt.%) and the formation of composites causes the appearance of more pronounced surface reliefs. Introducing 0.5 wt.% of SWCNTs (Figure 3b) facilitates the formation of fibers with smooth surfaces. It should be noted that the addition of precisely this amount of SWCNTs (0.5 wt.%) improves the mechanical characteristics of the composite fibers as compared to those of other samples containing 0.05 and 2.5 wt.% of nanotubes (Figure 4). The microphotograph of the fiber containing 2.5 wt.% of SWCNTs clearly shows the presence of oriented structural elements (appearing due to the increased content of isometrical SWCNT particles).

The fibers exposed to the lysozyme solution for 4 weeks retained their sizes and shapes. However, the surface reliefs of the fibers became more pronounced, and fibrillation of the surfaces occurred. The fibers modified with 0.05 wt.% of SWCNTs contain well-oriented structural elements on their surfaces. It should be noted that an increase in SWCNT content led to surface relief sharpening upon exposure to the biologically active medium; the formation of longitudinal pores and cracks is observed (Figure 3e–g). These changes in the surface structures of the composite fibers after exposure to the lysozyme solution are indicative of chitosan resorption that began from the fiber surfaces.

### 3.2. IR Spectroscopy

The prepared pure chitosan and chitosan-based composite fibers were characterized via FTIR spectroscopy. The spectra obtained contain several characteristic bands of different intensities. The wide band in the 3500–3200 cm^−1^ region is assigned to O-H stretching vibrations (hydroxyl groups involved in the formation of hydrogen bonds); the bands at 3360 and 3290 cm^−1^ are related to N-H stretching vibrations of amino groups; the bands at 2920 and 2865 cm^−1^ are assigned to C-H stretching vibrations; the band at 1650 cm^−1^ is assigned to stretching vibrations of C=O in the N-acyl fragment (Amide I); the peak at 1585 cm^−1^ is related to N-H bending vibrations (Amide II). The bands near 1370 and 1320 cm^−1^ are attributed to C-N stretching vibrations (Amide III) of acylated and deacylated fragments of glucosamine units. Several intense peaks near 1025 cm^−1^ are attributed to the stretching vibrations of C-O (C-OH) bonds of numerous hydroxyl groups; the band of moderate intensity near 890 cm^−1^ corresponds to C-O (C-O-C) vibrations of the O-glycoside bond.

**Figure 5:**
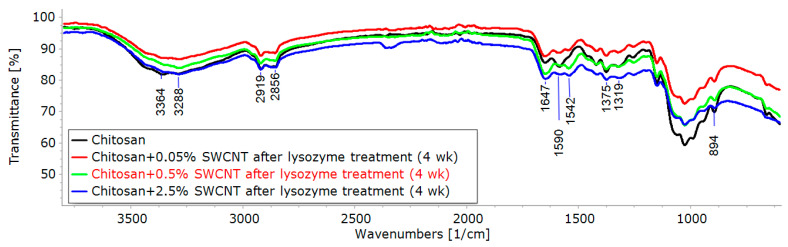
FTIR spectra of chitosan fiber and composite fibers containing 0.05%, 0.5%, and 2.5 wt.% SWCNTs.

The treatment of the chitosan and composite fibers with a solution of lysozyme (an enzyme of glycosidase family (EC 3.2.1.17)) resulted in the hydrolytic cleavage of the O-glycoside bond in chitosan, which was confirmed via FTIR spectroscopy (a significant decrease in the relative intensity of the band related to the C-O vibrations of the O-glycoside bond). It should be noted that prolonged exposure to the lysozyme solution (Figure 4) and an increase in the SWCNT content in the composite fiber (Figure 5) led to the amplification of this effect and an increase in the degree of hydrolysis of the chitosan. It can be assumed that the presence of carbon tubes in the fibers led to the orientation of chitosan macromolecules on their surfaces, as shown in [56], for chitosan macromolecules and chitin nanofibrils.

Also of note is the appearance of a new band at 1737 cm^−1^ with an increasing degree of chitosan hydrolysis. This band may be attributed to the vibrations of C=O bonds in the aldehyde groups that appear upon the opening of semi-acetal rings of terminal glucosamine units (whose concentrations increase with increasing degrees of hydrolysis).

### 3.3. Measurements of Strength and Deformation Characteristics

**Figure 6:**
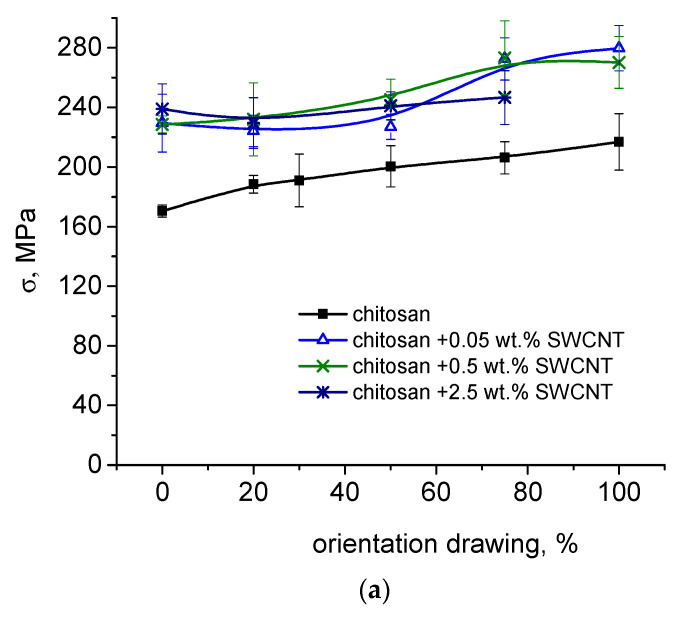

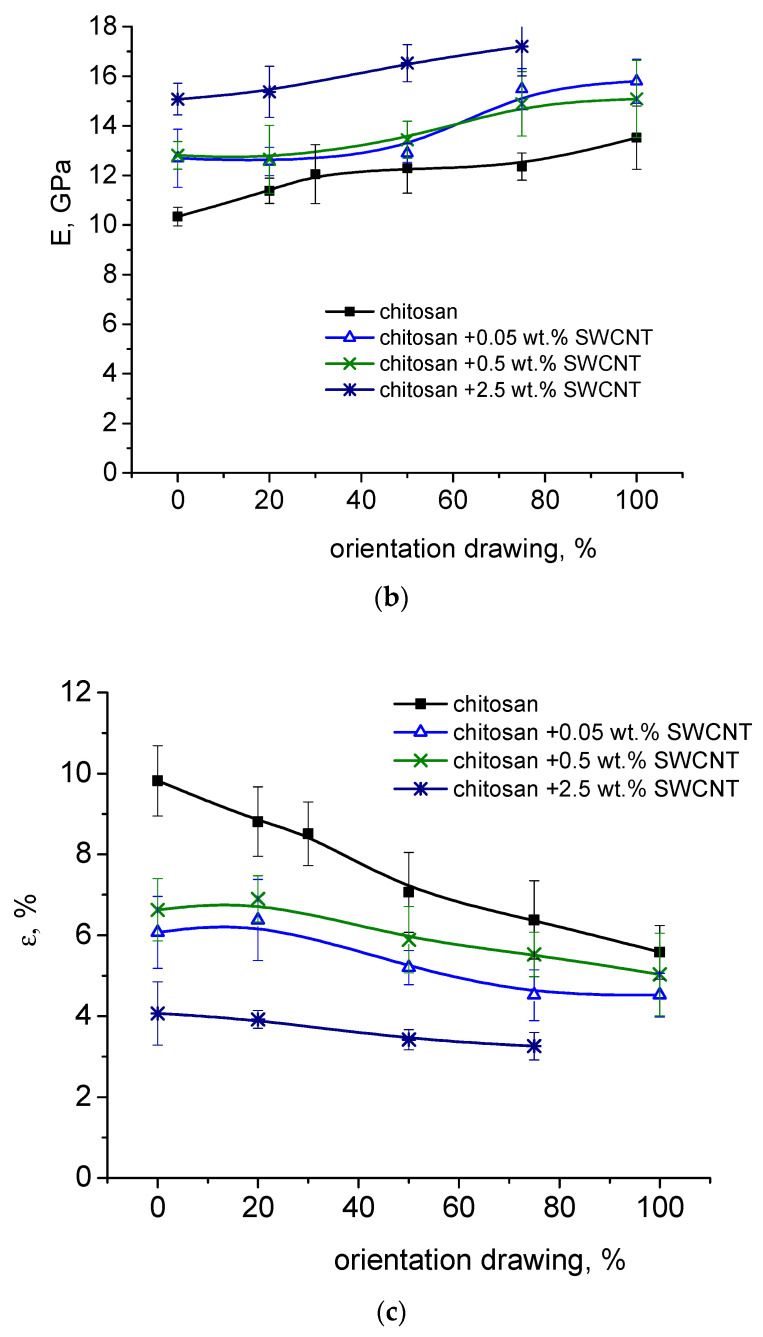
Dependences of tensile strength, σ (**a**), Young’s modulus, E (**b**), and elongation at break, ε (**c**) on the degree of orientational drawing for pure chitosan fibers and SWCNT-containing composite fibers.

The dependences of the mechanical characteristics of the composite fibers containing 0.05, 0.5, and 2.5 wt. % of SWCNTs on the degree of orientational drawing are provided in Figure 6a–c.

The data presented in Figure 6 imply that the introduction of nanotubes into the chitosan scaffold exerts a significant influence on the strength, elasticity, and deformation properties of the composite fibers.

The strength of the composite fibers increases by 50%, and their elastic modulus increases by 50% when compared to the corresponding parameters of the pure chitosan samples. For all composites (SWCNT contents 0.05–0.5 wt.%), the tensile strength and elastic modulus values increase monotonically with increasing degrees of orientational drawing; the dependences of the tensile strength and modulus values of the composite fibers on the degree of drawing are similar to those of pure chitosan fibers. Introducing SWCNTs into the chitosan scaffold facilitates the additional orientation of chitosan macromolecules, which increases the strength. A similar effect was observed upon the addition of chitin nanofibrils into chitosan fiber [56]. When a higher amount (2.5 wt.%) of filler is introduced, the behavior of the fiber strength changes: this parameter virtually does not depend on the degree of orientational drawing. This is related to the high content of single-walled carbon nanotubes in the composite solution and the formation of a rigid SWCNT framework. These fibers have high elastic modulus values (17.2 ± 1.2 GPa). The maximum orientational drawing of the fibers was equal to 75%.

The introduction of single-walled carbon nanotubes causes significant deteriorations in the deformation characteristics of the composite fibers; the tensile strain virtually does not change with increasing degrees of fiber drawing, unlike the corresponding characteristics of pure chitosan fibers. The tensile strain of the pure chitosan fiber obtained at λ = 100% is almost similar to that of the fibers containing 0.05–0.5 wt.% SWCNTs, although the tensile strength and elastic modulus values of the composite fibers are greater than those of pure chitosan fibers by 30%.

### 3.4. Measurements of Electrical Resistivity of Fibers

**Figure 7:**
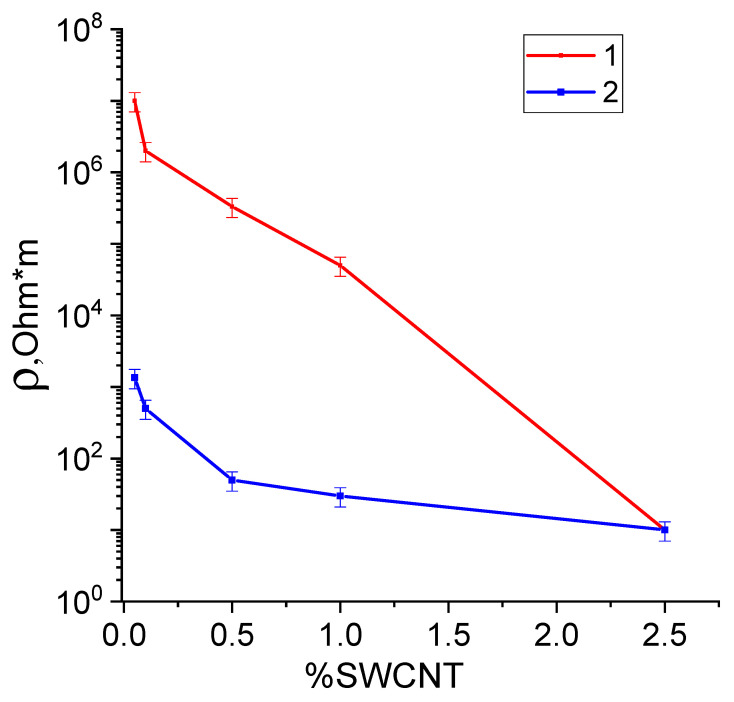
Dependences of specific volume resistivities of chitosan-based composite fibers on the SWCNT content: the initial chitosan and composite fibers (curve 1) and the fibers kept for 24 h at a relative humidity of 98% (curve 2).

Figure 7 presents the dependences of the specific resistivities of the composite fibers containing 0.05, 0.1, 0.5, 1,0, and 2.5 wt. % of SWCNTs on the filler concentration. The experiments involved the initial fibers and the fibers kept in a desiccator at a relative humidity of 98% for 24 h.

As can be seen from Figure 7, introducing SWCNTs into chitosan fiber results in a significant (by six orders of magnitude) decrease in its resistivity.

Pure chitosan fiber possesses ionic conductivity, and its resistivity is equal to 108–109 Ohm·m, which coincides with the data reported in [57]. When the chitosan scaffold contains carbon nanotubes, the composite demonstrates both ionic and electronic conductivities, which cause a decrease in the specific resistivity value. The presence of isometric conducting SWCNT particles among uniaxially oriented chitosan macromolecules promotes the formation of a conducting cluster even at small (lower than 2.5 wt.%) contents of SWCNTs [58,59]. The formation of conducting clusters also enhances the conductivity of the composite fibers.

After the exposure of pure chitosan fibers in a desiccator at a humidity of 98%, sharp decreases in their electrical resistivity values are observed (Figure 7, curve 2). It is known that chitosan is a hydrophilic polymer [60], and an increase in the content of conducting elements (adsorbed dissociated water molecules) results in an increase in the ionic conductivity of a fiber. Introducing SWCNTs causes a decrease in resistivity due to the formation of the conducting cluster and an increase in the electronic component of conductivity.

**Figure 8:**
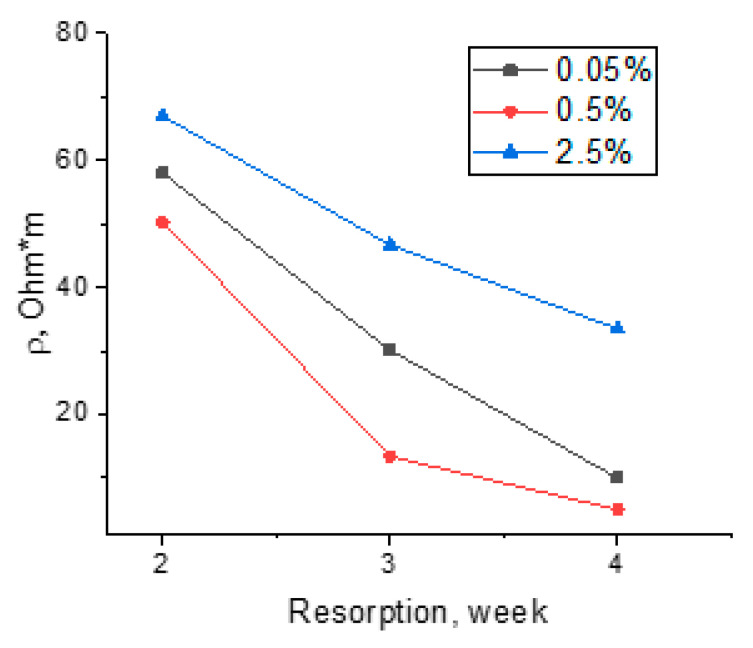
Dependences of specific volume electrical resistivities of chitosan-based composite fibers containing 0.05, 0.5, and 2.5 wt. % SWCNTs on bioresorption time.

The dependences of the electrical conductivities of the composite fibers on the time of exposure to the lysozyme-containing buffer solution were obtained (Figure 8). It can be seen that the resistivities of the composite fibers decrease with increasing exposure time.

This is explained by the fact that chitosan fibers are hydrophilic [60] and become saturated with electrolyte ions. Additionally, chitosan contains ionogenic–NH3+ groups. In an electrolyte solution, ionogenic groups become hydrated, and chitosan becomes an ionic type of conductor. The higher the SWCNT content, the higher the contribution of the electronic current compared to that of the ionic current. Thus, the conductivity of the composite fiber containing 2.5 wt.% of SWCNTs changes insignificantly when increasing the time of exposure to the lysozyme-containing buffer solution because the fiber contains a dense network of conducting carbon nanotubes. The changes in electrical conductivity are determined by the amount of SWCNTs and their distribution in the scaffold. Thus, the saturation of composite fibers with electrolyte ions during their exposure to a lysozyme solution led to the formation of an ionic–electronic type of conductivity and a decrease in volume resistivity. The mechanical tests and scanning electron microscopy results show that the optimal SWCNT concentration is equal to 0.5 wt.%, which is confirmed by the high values of electrical conductivity (Figure 8). According to the SEM data (Figure 3), the homogeneous structure of the fiber facilitates an increase in electrical conductivity.

### 3.5. Investigation of the Biocompatibility of the Fibers via the MTT Technique

When designing composite materials with improved physical and chemical properties, one must bear in mind that any additional component can influence the biocompatibility of the resulting material. Carbon nanotubes are promising additives for the preparation of materials with improved properties. Soon after their discovery in 1991 [61], these nanosized materials received significant attention from experts in various fields of science, including materials science and biomedical engineering. Although carbon nanotubes possess unique properties and thus impart improved mechanical, electrical, and thermal properties to composite materials [62,63,64], the use of these materials for medicinal purposes is still open to question. On one hand, the carbon nanotubes in the initial state in the cultural medium showed cytotoxicity to certain cell lines, which may be related to the use of metal catalysts (such as nickel) in the preparation of the carbon nanotubes [65]. On the other hand, the carbon nanotubes introduced into composite materials showed high levels of biocompatibility [66,67,68]. The number of publications focused on the investigation of the interactions between carbon nanotubes and cell lines increases every year [52,69,70].

**Figure 9:**
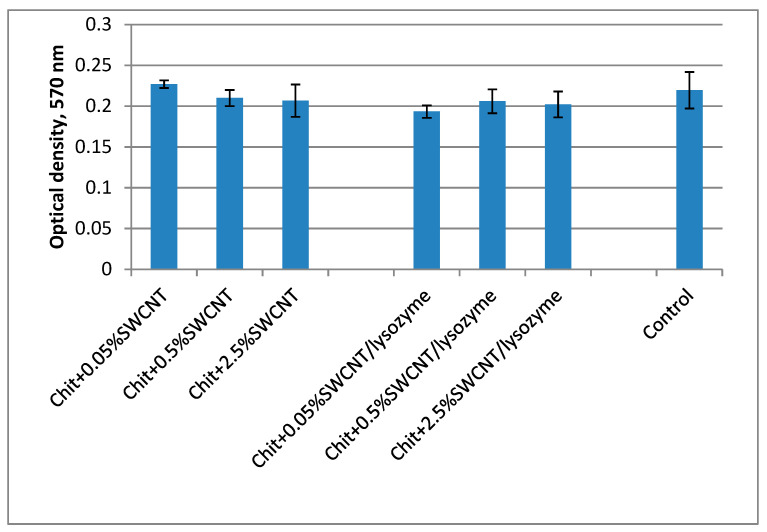
MTT test of FetMSC cells cultivated in the nutrient medium after incubation of the following fiber samples: Chit + 0.05%SWCNT—chitosan fibers containing 0.05% SWCNT; Chit + 0.5%SWCNT—chitosan fibers containing 0.5% SWCNTs; Chit + 2.5%SWCNT—chitosan fibers containing 2.5% SWCNTs; Chit + 0.05%SWCNT/lysozyme—chitosan fibers containing 0.05% SWCNTs after exposure to lysozyme solution for 4 weeks; Chit + 0.5%SWCNT/lysozyme—chitosan fibers containing 0.5% SWCNTs after exposure to lysozyme solution for 4 weeks; Chit + 2.5%SWCNT/lysozyme—chitosan fibers containing 2.5% SWCNTs after exposure to lysozyme solution for 4 weeks.

As previously mentioned, the presence of SWCNTs in chitosan fibers significantly improves their structural, mechanical, and conducting properties, but during exposure to a lysozyme solution, the emergence of SWCNTs on the fibers’ surfaces is possible. Since the literature contains contradictory data [65], it is necessary to study the cytotoxicity of the obtained composite fibers containing SWCNTs. Our studies have shown that the nutrient medium obtained after 4 days of incubating of the initial composite fiber has no toxic effect on human MSC cells (Figure 9). The number of viable cells in these samples is virtually similar to that in the control sample (the cells cultivated in the standard nutrient medium). An insignificant decrease in the number of viable cells in the case of the sample containing 0.05 wt.% of SWCNTs may be related to a partial release of nanotubes into the nutrient medium. The viability parameters of the cells in the nutrient medium samples in which the fibers with 0.5 and 2.5 wt.% of SWCNTs were kept are similar to the viability parameters of the control sample. As previously noted, it is possible that upon reaching a certain concentration, SWCNTs form a network that prevents the release of individual nanotubes into the nutrient medium.

### 3.6. Morphological Analysis

**Figure 10:**
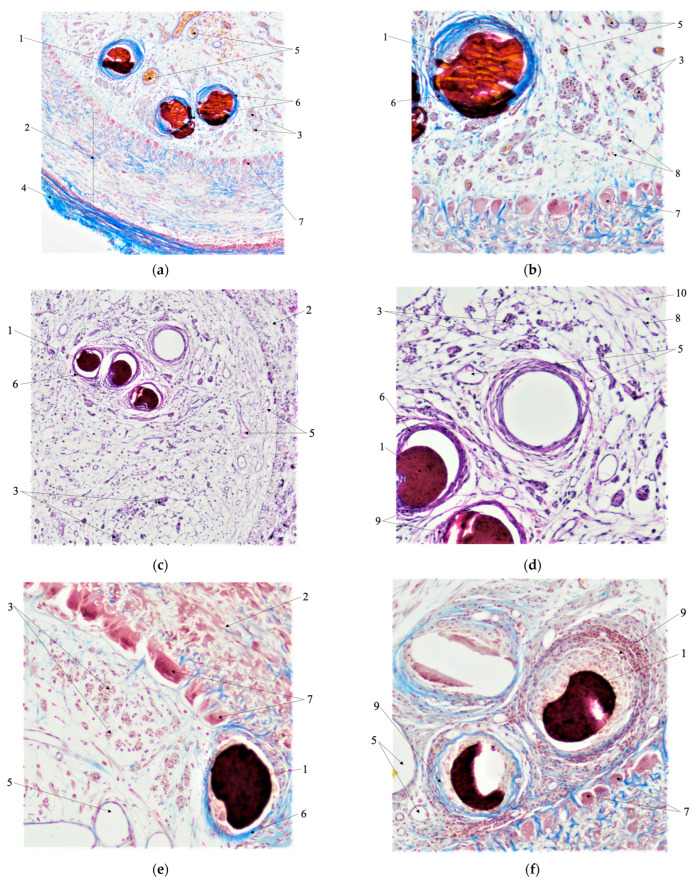
Transverse distal cuts of the scaffold with composite fibers containing 0.05 wt.% SWCNTs (**a**,**b**); 0.5 wt.% SWCNTs (**c**,**d**); 2.5 wt.% SWCNTs (**e**,**f**): 1—composite fibers containing SWCNTs, 2—scaffold, 3—nerve fibers, 4—connective tissue around the scaffold, 5—blood vessels, 6—connective tissue around composite fibers, 7—multinucleated foreign body giant cells, 8—fibroblasts, 9—leucocytes, and 10—collagen fibers.

Figure 10 presents the results of the morphological analysis of the nerve fibers with various artificial guides.

Figure 10 clearly shows the formation of multinucleated foreign body giant cells (FBGC) (arrow 7) in all experiments. These cells form a dense ring along the internal wall of the polylactide scaffold (arrow 2). Additionally, fibroblasts (arrow 8) and a moderate amount of collagen fibers (arrow 10) are localized in the bulk of the scaffold between the chitosan monofibers (arrow 1). This indicates the occurrence of bioresorption processes. Connective tissue fills the lumen of the polylactide scaffold, and growing nerve fibers (arrow 3) are located between connective tissue fibers. Furthermore, the outer surface of the scaffold is coated with connective tissue (arrow 4).

All monofilaments (arrow 1) become encapsulated, localized inside the scaffold eccentrically, and surrounded by connective tissue (arrow 6). This connective tissue is penetrated with numerous thin-walled vessels (arrows 5). It is noteworthy that no active bioresorption of monofibers is observed.

Upon implantation, chitosan monofibers containing 0.05 wt.% of SWCNTs (Figure 10a,b) become deformed due to compressive forces from the surrounding tissues. However, no signs of acute inflammatory reaction were observed after the implantation of chitosan monofibers containing 0.5 wt.% of SWCNTs (Figure 10c,d). These monofibers are surrounded by connective tissue (arrow 6) with moderate amounts of leukocytes (arrow 9).

In contrast, the implantation of chitosan monofibers containing 2.5 wt.% of SWCNTs (Figure 10e,f) results in much greater amounts of leukocytes (arrow 9) and multinucleated foreign body giant cells (FBGC) (arrow 7), indicating the presence of pronounced chronic aseptic inflammation.

## 4. Conclusions

Conducting, biocompatible, composite fibers based on chitosan and SWCNTs were developed. It was demonstrated that the specific volume resistivity of the composite samples decreased from 10^7^ to 1 Ohm*m when increasing the SWCNT content from 0.05 to 2.5 wt.%.

It was established that the presence of high amounts (2.5 wt.%) of carbon nanotubes (sources of electrical signals) in the composite fibers caused pronounced chronic, aseptic inflammation, although it did not result in a considerable increase in the number of growing nerve fibers.

It was found that the implanted chitosan monofibers containing 0.05 wt.% of SWCNTs became deformed under the action of compressive forces from the surrounding tissues, which led to the formation of a dense connective tissue capsule around the fiber.

The experiments showed that a conduit’s best variant of guiding fibers is the composite fiber containing 0.5 wt.% of SWCNTs. The addition of 0.5 wt.% of SWCNTs into composite fibers facilitates the formation of a denser fiber structure, which results in improved strength (σ = 260 MPa) and elastic (E = 14 GPa) characteristics. According to the results of the cytological studies, this composite fiber is biocompatible, it is not deformed in the process of in vivo studies, and it does not cause a pronounced inflammatory reaction. A thin connective tissue capsule is formed around the fiber.

## Data Availability

Not applicable.
